# Inhibition of JAK2 Suppresses Myelopoiesis and Atherosclerosis in *Apoe*^*−/−*^ Mice

**DOI:** 10.1007/s10557-020-06943-9

**Published:** 2020-02-21

**Authors:** Yang Tang, Wenli Liu, Wei Wang, Trevor Fidler, Britany Woods, Ross L. Levine, Alan R. Tall, Nan Wang

**Affiliations:** 1 Division of Molecular Medicine, Department of Medicine, Columbia University Medical Center, 630 W. 168th Street, New York, NY 10032 USA; 2 Department of Hematology, The First Hospital of Jilin University, Changchun, People’s Republic of China; 3 Human Oncology and Pathogenesis Program, Memorial Sloan Kettering Cancer Center, New York, NY USA; 4 Leukemia Service, Department of Medicine, Memorial Sloan Kettering Cancer Center, New York, NY USA

**Keywords:** JAK2 inhibitor, TG101348 (Fedratinib), Atherosclerosis, Myelopoiesis

## Abstract

**Objective:**

Increased myelopoiesis has been linked to risk of atherosclerotic cardiovascular disease (ACD). Excessive myelopoiesis can be driven by dyslipidemia and cholesterol accumulation in hematopoietic stem and progenitor cells (HSPC) and may involve increased signaling via Janus kinase 2 (JAK2). Constitutively activating JAK2 mutants drive biased myelopoiesis and promote development of myeloproliferative neoplasms (MPN) or clonal hematopoiesis, conditions associated with increased risk of ACD. JAK2 inhibitors have been developed as a therapy for MPNs. The potential for JAK2 inhibitors to protect against atherosclerosis has not been tested. We therefore assessed the impact of JAK2 inhibition on atherogenesis.

**Methods:**

A selective JAK2 inhibitor TG101348 (fedratinib) or vehicle was given to high-fat high-cholesterol Western diet (WD)–fed wild-type (WT) or *Apoe*^*−/−*^ mice. Hematopoietic cell profiles, cell proliferation, and atherosclerosis in WT or *Apoe*^*−/−*^ mice were assessed.

**Results:**

TG101348 selectively reversed neutrophilia, monocytosis, HSPC, and granulocyte-macrophage progenitor (GMP) expansion in *Apoe*^*−/−*^ mice with decreased cellular phosphorylated STAT5 and ERK1/2 and reduced cell cycling and BrdU incorporation in HSPCs, indicating inhibition of JAK/STAT signaling and cell proliferation. Ten-week WD feeding allowed the development of marked aortic atherosclerosis in *Apoe*^*−/−*^ mice which was substantially reduced by TG101348.

**Conclusions:**

Selective JAK2 inhibition reduces atherogenesis by suppressing excessive myelopoiesis in hypercholesterolemic *Apoe*^*−/−*^ mice. These findings suggest selective JAK2 inhibition as a potential therapeutic approach to decrease ACD risk in patients with increased myelopoiesis and leukocytosis.

**Electronic supplementary material:**

The online version of this article (10.1007/s10557-020-06943-9) contains supplementary material, which is available to authorized users.

## Introduction

Atherosclerosis is a lipoprotein-driven chronic inflammatory disease of the arterial wall [1]. Myeloid cells including monocytes, macrophages, neutrophils, and dendritic cells are critical players in innate and acquired immunity that are also responsible for the initiation and progression of atherosclerosis. It is generally accepted that a major proportion of myeloid cells in atherosclerotic plaques are recruited from circulating blood cells. Leukocytosis is associated with increased all-cause mortality [2, 3], due in large part to the increased morbidity and mortality of ischemic atherosclerotic cardiovascular diseases (ACD) [3, 4]. While leukocytosis could be a marker of other processes such as infection, there is considerable evidence that leukocytosis directly promotes the entry of monocytes and neutrophils into the arterial wall, increasing atherosclerosis and thrombosis [4].

Dyslipidemia and accumulation of cholesterol in hematopoietic stem and progenitor cells (HSPC) may link increased HSPC proliferation with myeloid bias to atherogenesis. Mice deficient in the adenosine triphosphate-binding cassette (ABC) transporters A1 and G1 (ABCA1 and ABCG1), which promote cholesterol efflux from myeloid cells, developed monocytosis, neutrophilia, and expansion of HSPC [5]. A similar observation was made in Western diet (WD)–fed hypercholesterolemic *Ldlr*^*−/−*^ and *Apoe*^−/−^ mice [6], where monocytosis and neutrophilia were associated with HSPC expansion. Competitive BM transplantation experiments suggested a cell intrinsic proliferative advantage and biased myelopoiesis of *Apoe*^−/−^ or *Abca1*^−/−^*Abcg1*^−/−^ HSPCs relative to WT HSPCs giving rise to increased numbers of myeloid progenitors, monocytes, and neutrophils [5, 6]. In these studies, the cell intrinsic proliferative advantage of HSPCs was associated with increased cell surface levels of the common β subunit of the GM-CSF/IL-3 receptor (CBS) and increased GM-CSF/IL-3 signaling [5, 6]. Importantly, CBS deficiency in WD-fed *Apoe*^−/−^ mice reduced HSPC expansion, monocytosis, and neutrophilia and decreased atherogenesis, causally linking aberrant cell proliferation signaling in hematopoietic cells to excess myelopoiesis, monocytosis, neutrophilia, and atherogenesis [7].

Janus kinase 2 (JAK2) is a non-receptor tyrosine kinase and a critical node in multiple growth factor and cytokine-mediated signaling pathways regulating hematopoiesis, including the GM-CSF/IL-3 signaling cascade [8]. Loss of JAK2 is embryonically lethal in mice [9], due to defective hematopoiesis. Conditional knockout of *Jak2* primarily in hematopoietic cells in adult mice results in a marked defect in hematopoiesis and hematopoietic stem cell function [10, 11]. The critical role of JAK2 in myelopoiesis in humans is illustrated by *JAK2* mutations. Acquired activating mutations of *JAK2* in hematopoietic tissues, with *JAK2*^*V617F*^ as the most common, drive development of myeloproliferative neoplasms (MPNs) [12]. *JAK2*^*V617F*^ is also one of several genetic variants that drive clonal hematopoiesis of indeterminate potential (CHIP) which is common in the elderly [13, 14]. Both *JAK2*^*V617F*^-driven MPN and CHIP are associated with increased risk of CVD [15, 16].

Subsequent to the discovery of *JAK2*^*V617F*^ as the major mutation that drives MPN, JAK2 inhibitors were developed for MPN therapy. Ruxolitinib was the first JAK1/2 inhibitor that has been approved for the treatment of intermediate or high-risk myelofibrosis [17], including primary myelofibrosis, post-polycythemia vera myelofibrosis, and post-essential thrombocythemia myelofibrosis. While JAK2 inhibition ameliorates MPN phenotypes driven by JAK2 or related gene mutations in both mouse models and humans, its impact on excessive myelopoiesis and atherogenesis caused by hypercholesterolemia and defective cholesterol efflux from HSPCs and myeloid progenitors is unknown. In this study, we have assessed the impact of treatment with TG101348 (fedratinib), a selective inhibitor of JAK2 [18, 19], on myelopoiesis and atherosclerosis in WD-fed *Apoe*^−/−^ mice.

## Materials and Methods

### Mice

WT (C57BL/6) and *Apoe*^*−/−*^ (B6.129P2-APOE<tm1 Unc>/J) female mice were purchased from the Jackson Laboratory. At 8 weeks of age, all mice were fed a WD (21% milk fat, 0.2% cholesterol; catalog no. TD88137; Harlan Teklad) for the specified period of time. One week after WD feeding, *Apoe*^*−/−*^ mice and WT mice received TG101348 in phosphate-buffered saline (PBS) containing 0.5% methyl-cellulose (Sigma) or vehicle (PBS containing 0.5% methyl-cellulose) via oral gavage, at a dose of 120 mg/kg per day in the first week, and 240 mg/kg per day in the following days. Where appropriate, mice will be euthanized by CO_2_ asphyxiation followed by cervical dislocation, a method consistent with the recommendations of the Panel on Euthanasia of the American Veterinary Medical Association. All animal experiments and procedures were designed according to NIH guidelines and approved by Columbia University IACUC.

### Complete Blood Count Analysis

Complete blood count analysis was performed using freshly drawn blood via angular vein and the FORCYTE Veterinary Analyzer (Oxford Science Inc.).

### Flow Cytometry

For identification of monocytes and neutrophils from whole blood, we used the following strategy. Blood was drawn via angular vein collected into EDTA tubes, which were immediately placed on ice. RBCs were lysed (BD pharm Lyse; BD Biosciences), and leukocytes were pelleted by centrifugation, and resuspended in HBSS (0.1% BSA *w*/*v*, 5 mM EDTA). Cells were stained with a cocktail of antibodies against CD45-pacific blue, Ly6-C/G-PerCP-Cy5.5 (BD Biosciences), CD115-APC, and CD11b-PEcy7(eBioscience). Samples were analyzed on an LSR-II (BD Biosciences). Neutrophil were defined as CD45^hi^ Ly6-C/G^hi^ CD115^lo^. Monocytes were identified as CD45^hi^CD115^hi^ and further subdivided into Ly6-C^hi^ and Ly6-C^lo^ [20, 21].

For BM hematopoietic cell profiling, BM cells were stained and analyzed as previously described [22]. Briefly, BM cells from mouse femurs and tibias were stained with a cocktail of antibodies to lineage-committed cells (CD45R, CD19, CD11b, CD3e, TER-119, CD2, CD8, CD4, and Ly-6C/G, all FITC conjugated; Bioscience), Sca 1-pacific blue and c-Kit-BV605 to identify HSPC (Lin^−^ Sca1^+^ c-Kit^+)^ cells and HSPCs (Lin^−^ Sca1^−^ c-Kit^+^) together with antibodies against CD16/CD32 (FcγRII/III)-BV510 and CD34-APC to separate CMP (Lin^−^ Sca1^−^ c-Kit^+^, CD34^int^, FcγRII/III^int^), granulocyte-macrophage progenitor (GMP) (Lin^−^ Sca1^−^ c-Kit^+^, CD34^int^, FcγRII/III^hi^), and MEP (Lin^−^ Sca1^−^ c-Kit^+^, CD34^low^, FcγRII/III^low^). Where further identification of MEP population was required, ERP was defined as Lin^−^ Sca1^−^ c-Kit^+^, CD34^low^, FcγRII/III^low^, CD71^+^ CD41^−^, and MKP as Lin^−^ Sca1^−^ c-Kit^+^, CD34^low^, FcγRII/III^low^, and CD71^−^ CD41^+^. Cell cycle was quantified using 7-AAD. Phospho-flow was performed using antibodies to PE conjugated p-ERK1/2 or p-STAT5 [22, 23]. Briefly, BM cells were stained with antibodies described above and then fixed by 2% PFA in the room temperature for 10 min. The cells were then resuspended by Perm Buffer III (BD Biosciences) 30 min in the ice. After washing and resuspending by staining buffer, the PE Anti-ERK1/2(BD Phosflow) and PE Anti-STAT5(BD Phosflow) were added and incubated in room temperature for 30 min and then analyzed by flow cytometry.

### Proliferation Assays

Bone marrow cells were isolated from WD-fed WT or *Apoe*^*−/−*^ mice treated with vehicle or TG101348 (240 mg/day) for 6 weeks and then were cultured in IMDM (Invitrogen) with 10% FBS and 1 μM BrdU (Sigma). After 12-h incubation, cells were stained as described above (lineage changed to APC-conjugated and CD34 changed to PE-conjugated antibodies) for progenitor cells together with FITC-anti BrdU (Biolegend). Proliferation was quantified as percentage of BrdU + cells by flow cytometry.

### Atherosclerosis Study

The thoracic and abdominal descending aortas were collected from WD-fed *Apoe*^−/−^ mice following systemic perfusion with PBS by cardiac puncture of mice anesthetized with isoflurane vaporizer (inhale, ~ 5% isoflurane) and stained with Oil Red O, following fixation with 10% buffered formalin. Aortas were pinned in silicon dishes, and Oil Red O–positive areas were quantified using ImageJ software and expressed as the percentage of the total aorta area.

### Plasma Cholesterol Level Measurement

Plasma was collected from blood sample by centrifugation for 10 min at 10,000×*g* using a refrigerated centrifuge. Total cholesterol levels were measured using the Cholesterol E kit (Wako Diagnostics) as per the manufacturer’s instructions.

### Statistics

The number of mice used was estimated by power analysis based on the data from our previous studies. Normality assumption of the data distribution was assessed using Kolmogorov-Smirnov test. Data were analyzed by unpaired *t* test if data were normally distributed and two groups were involved. One-way ANOVA was used for more than two groups. Two-tailed analysis was performed in all statistical analyses. *p* value less than 0.05 was considered a significant difference.

## Results

### TG101348 Reverses Monocytosis and Neutrophilia in WD-Fed *Apoe*^*−/−*^ Mice

**Fig. 1:**
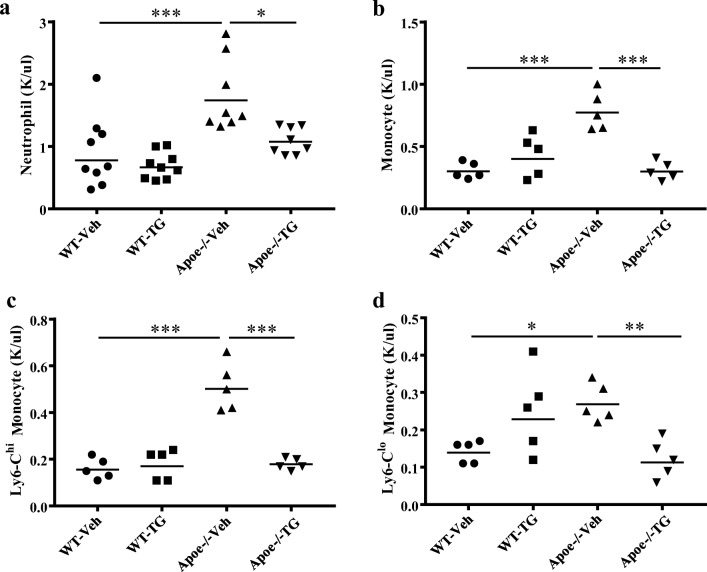
TG101348 selectively reverses monocytosis and neutrophilia in WD-fed *Apoe*^*−/−*^ mice. After 1-week WD feeding, female mice were treated with vehicle or TG101348 for 30 days and peripheral blood cell profiles were assessed as described in “Materials and methods.” **a** Neutrophil counts assessed by automated blood cell analyzer. **b** Total monocyte, **c** Ly6C^hi^, and **d** Ly6C^lo^ monocyte counts were determined with 5 randomly selected samples by flow cytometry in combination with automated blood cell analyzer. One-way ANOVA. Data are presented as mean ± SEM. *p < 0.05, **p < 0.01, ***p < 0.001

To assess the impact of TG101348 on hematopoiesis, we administered vehicle or TG101348 to WD-fed WT or *Apoe*^−/−^ mice at a dose that sustained the plasma concentration above the cellular IC_50_ and effectively reduced myelopoiesis, hematocrit, and leukocytosis in a murine model of MPN induced by hematopoietic *Jak2*^*V617F*^ expression [18]. Consistent with the previous report, TG101348 did not cause apparent abnormalities or toxicities in either WT or *Apoe*^−/−^ mice, with no difference in gain of body weight (Supplemental Figure 1A, B). WD feeding in combination with APOE deficiency markedly increased plasma total cholesterol levels, as expected. TG101348 did not affect plasma cholesterol or HDL levels (Supplemental Figure 1C, D). *Apoe*^−/−^ mice WD-fed for one week showed monocytosis and neutrophilia relative to the WD-fed WT mice (supplemental Figure 2A, B) and continued WD-feeding made this leukocytosis even more pronounced (Fig. 1), as reported [6, 24, 25]. WD feeding, relative to chow diet, moderately increases plasma cholesterol levels and has no effect on white blood cell counts including monocyte and neutrophil counts in WT mice [24]. At the time point just before vehicle or TG101348 treatment, the baseline levels of monocytes, neutrophils, platelets, and hemoglobin concentration showed no difference between vehicle and TG101348 groups (Supplemental Figure 2A, B; Supplemental Figure 3A, B). However, after 30 days of TG101348 treatment, the neutrophilia and monocytosis in *Apoe*^−/−^ mice was reduced to the level of the WT mice (Fig. 1a, b). Ly6C^hi^ monocytes preferentially enter atherosclerotic lesions where they differentiate to macrophages that are actively involved in progression of atherosclerosis [24, 25]. *Apoe*^−/−^ mice showed a marked increase in Ly6C^hi^ monocyte which was reversed by TG101348 (Fig. 1c, Supplemental Figure 5). TG101348 also reduced Ly6C^lo^ monocytes in *Apoe*^−/−^ mice (Fig. 1d). In contrast, TG101348 showed no effects on neutrophil and monocyte counts in the WT mice (Fig. 1a, b) nor did it affect platelet counts in either group (Supplemental Figure 3D). TG101348 slightly decreased hemoglobin levels and red blood cell counts in WT and *Apoe*^−/−^ mice (Supplemental Figure 3C and Supplemental Figure 4). These findings indicate that TG101348 selectively reverses monocytosis and neutrophilia in WD-fed *Apoe*^−/−^ mice.

### TG101348 Reverses HSPC Expansion in WD-Fed *Apoe*^*−/−*^ Mice

**Fig. 2:**
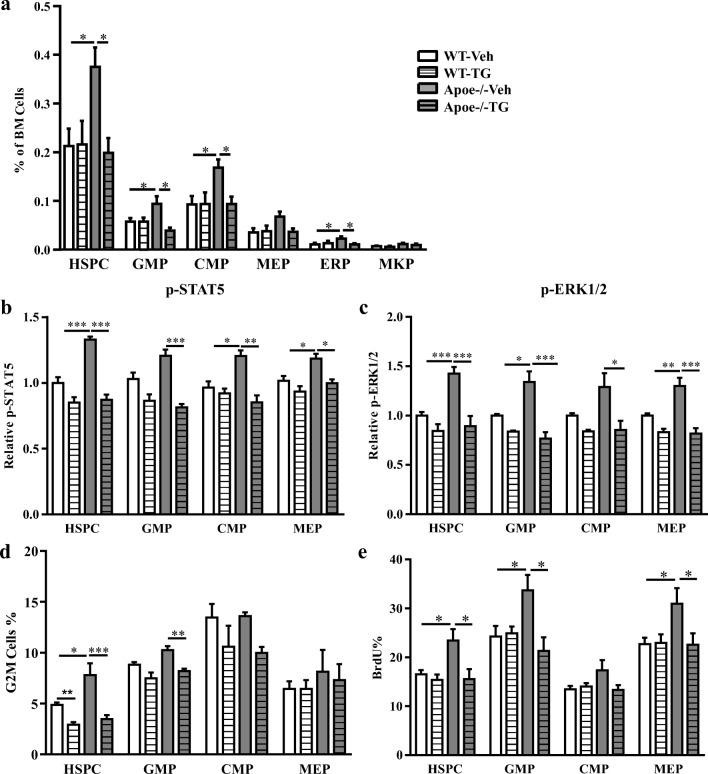
TG101348 reverses excessive proliferation and expansion of HSPCs in WD-fed *Apoe*^*−/−*^ mice. Female mice were treated with vehicle or TG101348 for 9 weeks. **a** Hematopoietic cell profile in bone marrow. Progenitor cells were defined as HSPC (Lin^−^ Sca1^+^ c-Kit^+^), CMP (Lin^−^ Sca1^−^ c-Kit^+^ CD34^int^FcγRII/III^int^), GMP (Lin^−^ Sca1^−^ c-Kit^+^ CD34i^nt^FcγRII/III^hi^), MEP (Lin^−^ Sca1^−^ c-Kit^+^ CD34^low^FcγRII/III^low^), ERP(Lin^−^ Sca1^−^ c-Kit^+^ CD34^low^FcγRII/III^low^CD71^+^ CD41^−^), and MKP (Lin^−^ Sca1^−^ c-Kit^+^ CD34^low^FcγRII/III^low^CD71^−^ CD41^+^) by flow cytometry. **b** Phospho-flow of p-STAT5 and **c** p-ERK1/2 relative MFI in HSPCs. **d** Percentage G2M phase positive cells of HSPCs. **e** HSPC proliferation was determined by BrdU incorporation. Four to six randomly selected sample per group were used for each of the assays. One-way ANOVA. Data are presented as mean ± SEM. *p < 0.05, **p < 0.01, ***p < 0.001. *n* = 4 (**e**) to 6 (**a**–**d**) mice per group

Previous studies indicate that neutrophilia and monocytosis in *Apoe*^−/−^ mice are associated with increased proliferation and expansion of multipotential progenitor cells (HSPCs) [6]. This HSPC expansion appears to be critical for aberrant myelopoiesis and leukocytosis in *Apoe*^−/−^ mice [6, 7]. To assess the impact of TG101348 on HSPC expansion and myelopoiesis, we analyzed bone marrow hematopoietic cell profiles. Consistent with previous findings [6], the HSPC population was expanded in *Apoe*^−/−^ mice fed WD for 10 weeks relative to the WT control (Fig. 2a) and so were common myeloid progenitors (CMP) and GMPs. TG101348 treatment in the last 9 weeks of WD feeding reversed the expansion of HSPCs and myeloid progenitors in *Apoe*^−/−^ mice but showed no effects on these cell populations in WT mice (Fig. 2a). WD-fed *Apoe*^−/−^ mice also showed splenomegaly (Supplemental Figure 6A, B), likely as a result of increased extramedullary hematopoiesis as reported [6]. TG101348 selectively reversed the splenomegaly in *Apoe*^−/−^ mice and showed no effect on spleen weight in WT mice (Supplemental Figure 6A, B).

Increased HSPC proliferation and expansion in *Apoe*^−/−^ mice reflect enhanced signaling in pathways involved in cell survival and proliferation, including IL-3/GM-CSF classical signaling pathways [6]. Indeed, levels of pSTAT5 and pERK1/2, the downstream signaling molecules of IL-3/GM-CSF pathways, in HSPCs from *Apoe*^−/−^ mice were increased (Fig. 2b, c) as assessed by phospho-flow cytometry, as reported [6], indicating increased proliferation signaling. pSTAT5 and pERK1/2 were also increased in GMPs and CMPs (Fig. 2b, c). Consistent with increased proliferation signaling, cell cycling, as assessed by G2M phase positive cells, and cell proliferation, as assessed by BrdU incorporation, in HSPCs from *Apoe*^−/−^ mice also was increased (Fig. 2d, e), as reported [6]. TG101348 reversed the increase in pSTAT5, pERK1/2, cell cycling, and BrdU incorporation in HSPCs and GMPs (Fig. 2b–e, Supplemental Figure 7A, B; Supplemental Figure 8). Together, these data suggest that TG101348 reverses HSPC expansion by inhibiting cell proliferation signaling that involves JAK2, STAT5, and ERK1/2.

### TG101348 Decreases Atherosclerosis in *Apoe*^*−/−*^ Mice

**Fig. 3:**
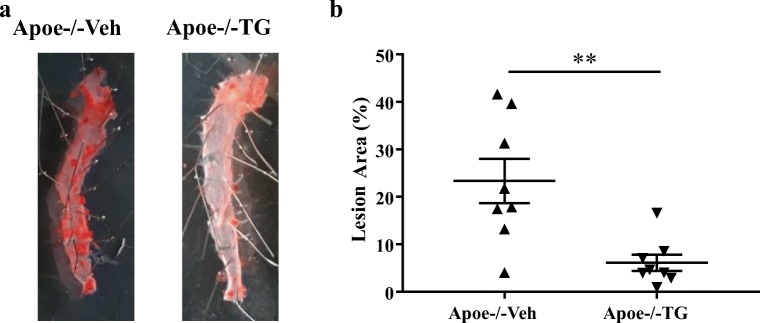
TG101348 suppressed atherosclerosis progression in *Apoe*^*−/−*^ mice. Female *Apoe*^*−/−*^ mice were fed WD for 1 week following by 9-week WD feeding with vehicle or TG101348 treatment. **a** Oil red O staining of aortas. **b** Plaque area as a percentage of total area. Unpaired *t* test. Data are presented as mean ± SEM. **p < 0.01

We next assessed the impact of TG101348 on atherogenesis in WD-fed *Apoe*^*−/−*^ mice. *Apoe*^*−/−*^ mice were fed WD for 10 weeks and treated with TG101348 or vehicle in the last 9 weeks as detailed in “Materials and methods.” TG101348 did not affect plasma total cholesterol levels following the treatment for 9 weeks (not shown), consistent with no change of HDL or non-HDL cholesterol levels following TG101348 treatment for 30 days (Supplemental Figure 1B). Atherogenesis was assessed by *en face* Oil Red O staining of the descending aorta. WD feeding induced marked Oil Red O stained and neutral lipid-rich aortic atherosclerosis in *Apoe*^*−/−*^ mice, and TG101348 substantially reduced atherosclerosis lesion area by ~ 74% (Fig. 3a, b). Together, these data indicate that TG101348 reduces atherogenesis in *Apoe*^−/−^ mice at least in part by selective suppression of HSPC expansion, excessive myelopoiesis, and leukocytosis.

## Discussion

Our studies demonstrate that TG101348 decreases atherosclerosis in *Apoe*^*−/−*^ mice, likely by selective inhibition of hematopoietic JAK2 that results in suppression of excessive myelopoiesis driven by enhanced cell proliferation signaling in HSPCs and myeloid progenitors and reversal of HSPC expansion and leukocytosis. This suggests the possible translation of TG101348 therapy in reducing the risk of ACD in association with moderate myeloproliferation with or without JAK2 mutations.

The clearest evidence for a role of excessive myelopoiesis in neutrophilia and monocytosis comes from studies of animal models with hypercholesterolemia and genetic deficiency in genes involved in cholesterol efflux from hematopoietic cells such as *Abca1*^*−/−*^/*Abcg1*^*−/−*^ or *Apoe*^*−/−*^ mice [5, 6]. However, excessive myelopoiesis and leukocytosis also occur in hypercholesterolemic mice such as long-term WD-fed *Ldlr*^*−/−*^ without deficiencies in cholesterol efflux genes [6]. *Ldlr*^*−/−*^ mice with loss of one copy of *Apoa1* also showed HSPC expansion, monocytosis, and neutrophilia [26]. Importantly, HDL-C levels in children with familial hypercholesterolemia showed an inverse correlation with monocyte counts [26]. Cell membrane cholesterol accumulation in HSPCs and myeloid progenitors increases surface CBS levels, leading to enhanced IL3- or GM-CSF-mediated cell proliferation signaling [5–7]. Although the precise mechanism responsible for the increased CBS expression at the cell surface remains elusive, hypercholesterolemia appears to disrupt a negative feedback desensitization response that downregulates CBS in response to IL3 or GM-CSF stimulation [27, 28]. Hypercholesterolemia-triggered neutrophilia and mobilization of Ly6C^hi^ monocytes from the spleen as a result of extramedullary hematopoiesis in WD-fed *Apoe*^*−/−*^ mice have been shown to promote atherogenesis [29, 30] and reversal of neutrophilia and reduction of Ly6C^hi^ monocytes by TG101348 likely contributes to reduced atherogenesis. Our studies demonstrate for the first time that selective inhibition of JAK2 reverses excessive myelopoiesis and reduces atherosclerosis in hypercholesterolemic mice.

Subsequent to the discovery of JAK2VF as a major mutation that drives development of MPN, JAK inhibitors were developed as a therapy for MPN. Following the approval of the JAK1/2 inhibitor ruxolitinib, TG101348 (fedratinib) was developed as a selective JAK2 inhibitor; clinical trials met the primary endpoint (the proportion of patients with a ≥ 35% reduction in spleen volume) with TG101348 for patients who had MPN and showed no response or intolerance to ruxolitinib [19]. Recently, the FDA has approved fedratinib for treatment of myelofibrosis [31]. TG101348 slightly decreased red blood cell counts and hemoglobin content in both WT and *Apoe*^*−/−*^ mice, consistent with anemia as a detected adverse effect of fedratinib in MPN therapy in clinical trials [32]. Other potential adverse effects of JAK inhibitors could include increases in LDL cholesterol and in body weight. However, since they may reflect amelioration of the underlying MPN [33, 34], they do not necessarily indicate an adverse effect on metabolic health.

Like many other tyrosine kinase inhibitors, inhibition of JAK2 by TG101348 is selective and the specificity is not absolute. TG101348 has been reported to inhibit FLT3 and RET, although with much higher IC_50_ relative to that of JAK2 [18]. FLT3 has a critical role in hematopoietic progenitor development and function [35]. In contrast to *Jak2*^*−/−*^, targeted disruption of *Flt3* results in normal, healthy, and fertile adult mice, in association with no morphological and quantitative changes of peripheral blood cells [36]. The only detectable impact of *Flt3*^*−/−*^ on mature blood cells in bone marrow was on lymphocytes, particularly reduction of B cells [36]. We did not detect any effect of TG101348 on lymphocytes in our models. Thus, we conclude that the primary impact of TG101348 on myelopoiesis in WD-fed *Apoe*^*−/−*^ mice is mediated by selective inhibition of JAK2, although minor effect mediated by FLT3 inhibition cannot be excluded.

In addition to MPNs, JAK inhibitors have been developed for therapy of other immune and inflammatory disorders such as rheumatoid arthritis and inflammatory bowel disease [37] reflecting the role of JAKs in regulation of type I and type II cytokine receptor–mediated signaling [37]. Atherosclerosis has long been recognized as a chronic inflammatory disease [1], and innate and adaptive immunities in the setting of hypercholesterolemia are a major contributor to development and progression of atherosclerosis [38]. Prominent examples include enhanced inflammasome activation and IL-1β production in atherosclerotic macrophages [39, 40]. The translational significance was demonstrated by the CANTOS trial showing that IL-1β antagonism decreases the risk of ACD [41, 42]. Enhanced inflammatory responses with increased production of pro-inflammatory cytokines and transcriptional and epigenetic reprogramming of HSPCs and myeloid progenitors in hypercholesterolemia or other settings of chronic systemic inflammation such as rheumatoid arthritis could lead to biased myelopoiesis and leukocytosis, increasing atherogenesis [43, 44]. This could form a vicious cycle with biased myelopoiesis/leukocytosis and chronic inflammatory responses strengthening and exacerbating each other. While not specifically tested in this study, it is possible that TG101348 reduces atherogenesis by suppressing type I and type II cytokine receptor–mediated pro-inflammatory responses in the setting of hypercholesterolemia in *Apoe*^*−/−*^ mice, in addition to reversal of leukocytosis, including acting directly on macrophages [45].

In summary, our studies suggest JAK2 inhibition as a novel therapeutic approach to decrease ACD risk associated with excessive myelopoiesis and leukocytosis.

## Electronic Supplementary Material

ESM 1 Supplemental Figure 1. (A-B) Body weight after and following 9 weeks of vehicle or TG101348 (8-10 mice/group). (C) plasma total cholesterol levels and (D) HDL-cholesterol following 30 days of vehicle or TG101348 treatment (8-10 mice/group). One-way ANOVA. Data are mean ± SEM. **p<0.01, ***p<0.001. Supplemental Figure 2. Baseline levels of (A) neutrophil or (B) monocyte counts in peripheral blood (at day 0 before vehicle or TG101348 treatment) were shown. One-way ANOVA. Data are mean ± SEM. *p<0.05, **p<0.01, ***p<0.001. Supplemental Figure 3. WT and *Apoe*^*-/-*^ mice hemoglobin and platelet counts. (A) The baseline hemoglobin concentration and (B) platelet counts in peripheral blood (at day 0 before vehicle or TG101348 treatment). (C) hemoglobin concentration and (D) platelet counts in peripheral blood following vehicle or TG101348 treatment for 30 days were shown. One-way ANOVA. Data are mean ± SEM. *p<0.05, **p<0.01, ***p<0.001. Supplemental Figure 4. Red blood cell counts following treatment with vehicle or TG101348 for 30 days. One-way ANOVA. Data are mean ± SEM. ***p<0.001. Supplemental Figure 5. Flow cytometry gating strategy of blood neutrophil and monocyte. Supplemental Figure 6. Decreased spleen weight in TG101348 treated Apoe-/- mice. Mice were fed WD for one week following by 9 weeks WD with vehicle or TG101348 treatment. (A) Absolute spleen weight and (B) spleen/body weight ratios. One-way ANOVA. Data are mean ± SEM. ***p<0.001. Supplemental Figure 7. Representative flow cytometric histograms of (A) p-STAT5 and (B) p-ERK1/2 in HSPC. Supplemental Figure 8. Flow cytometry gating strategy of hematopoietic progenitor cells. (PDF 414 kb)
