# Supplementary material for: Inhibition of JAK2 Suppresses Myelopoiesis and Atherosclerosis in Apoe−/− Mice
Source: Cardiovasc Drugs Ther. 2020 Feb 21;34(2):145–52. doi: 10.1007/s10557-020-06943-9 (PMC7125070; doi:10.1007/s10557-020-06943-9)

Supplemental Figure 1

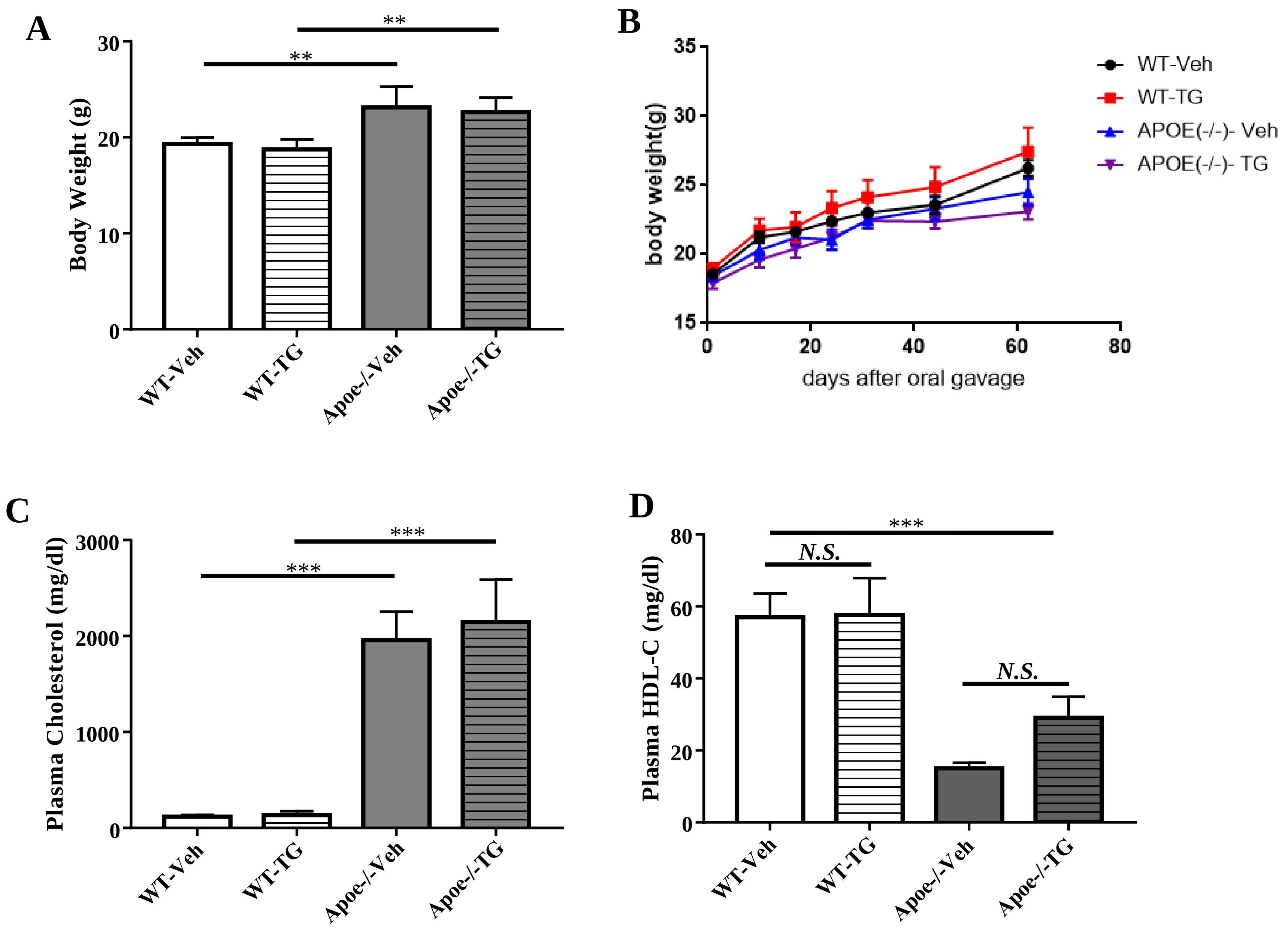

Supplemental Figure 2

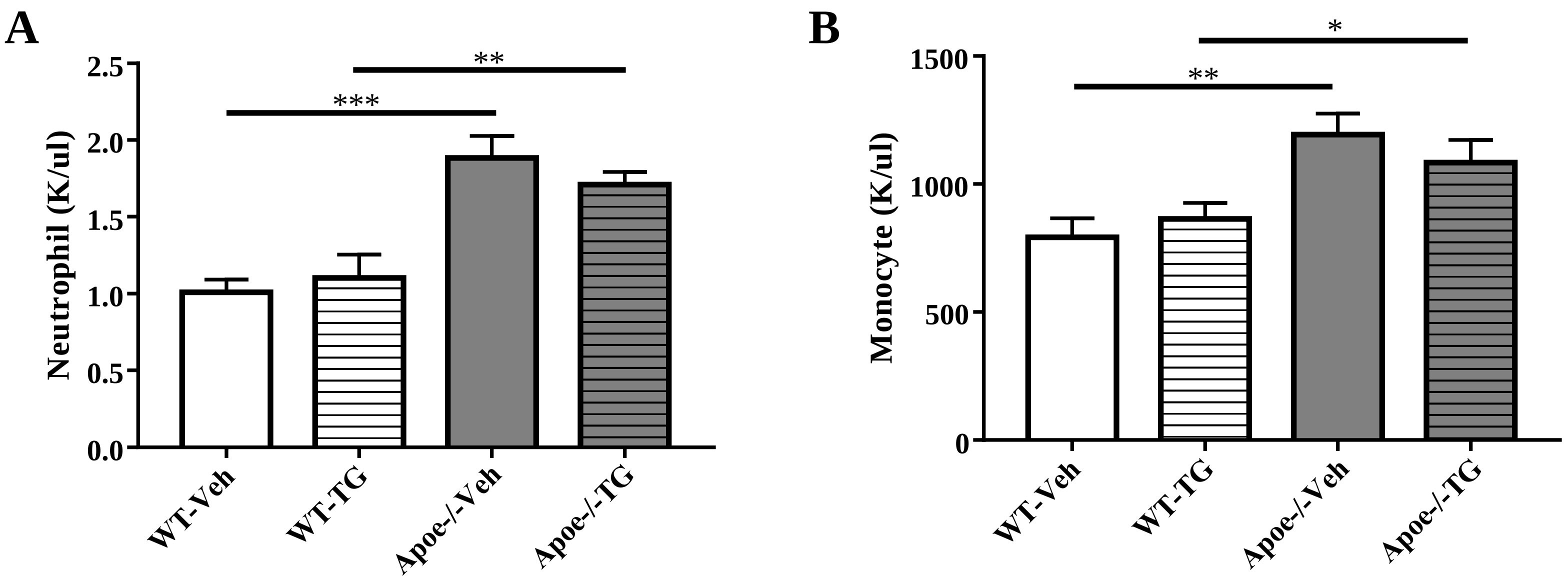

Supplemental Figure 3

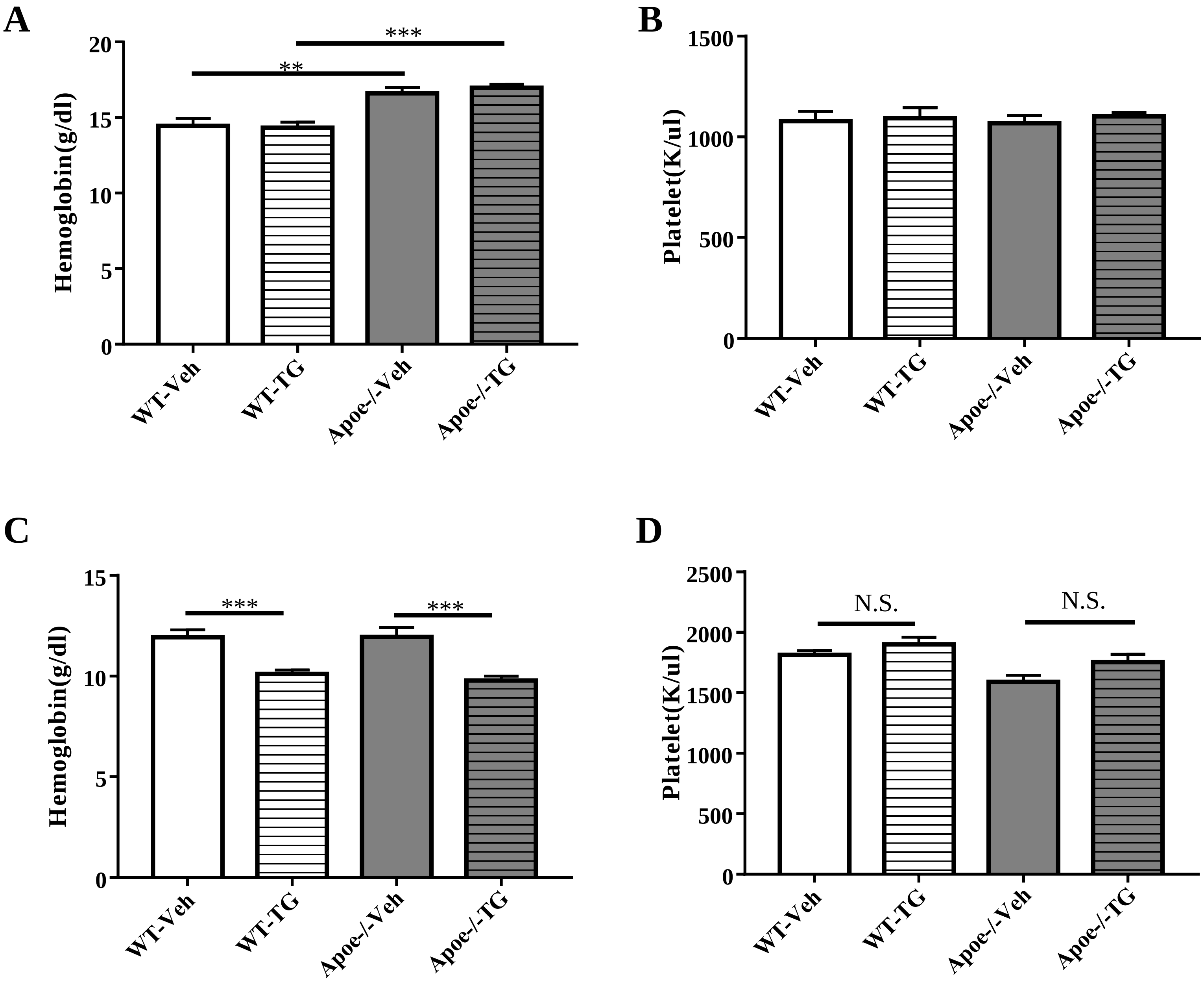

Supplemental Figure 4

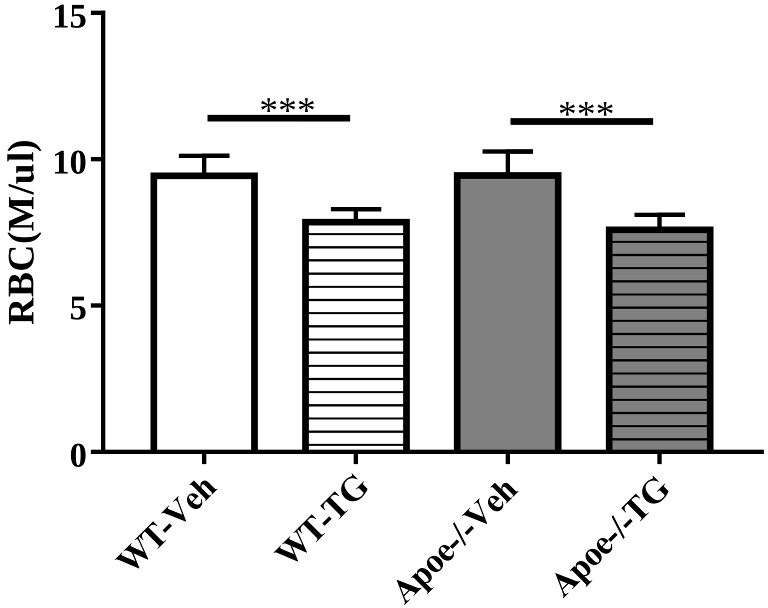

Supplemental Figure 5

Blood cells gating strategy

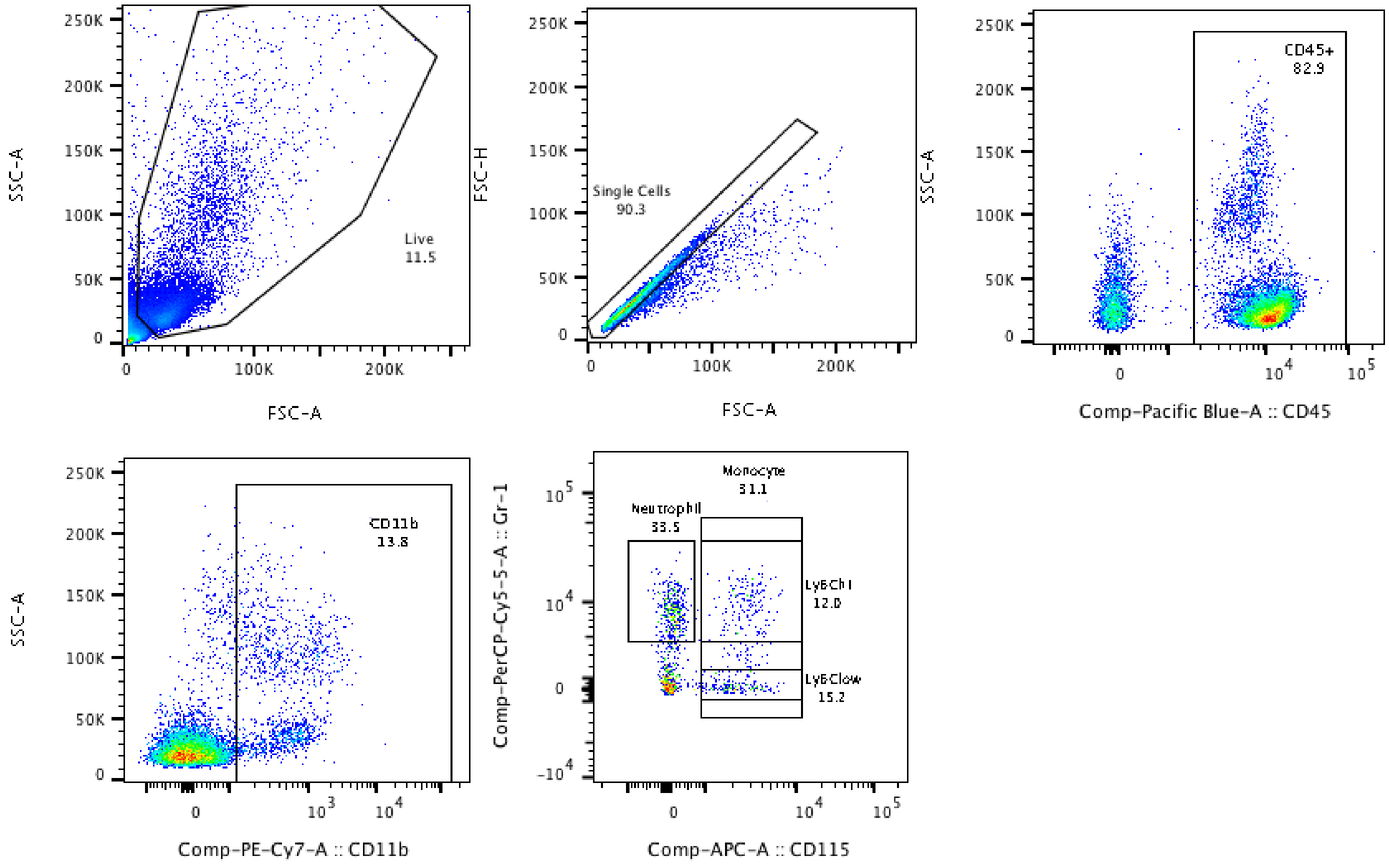

Supplemental Figure 6

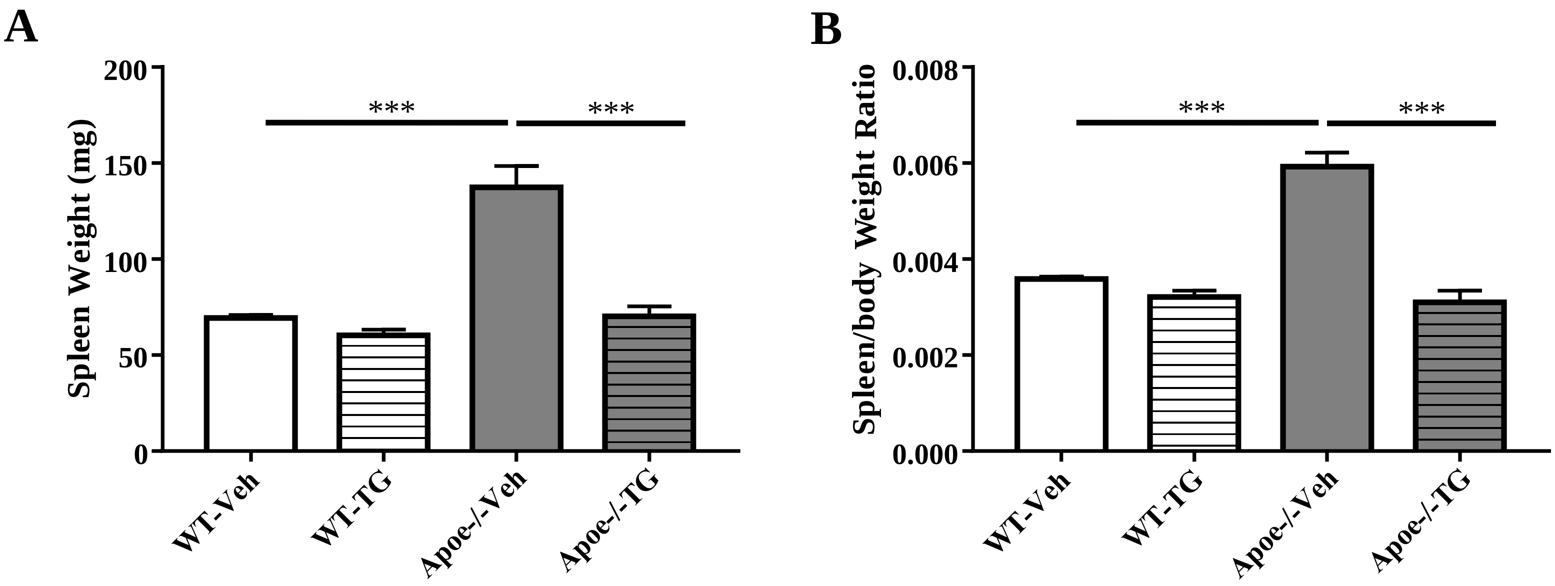

Supplemental Figure 7

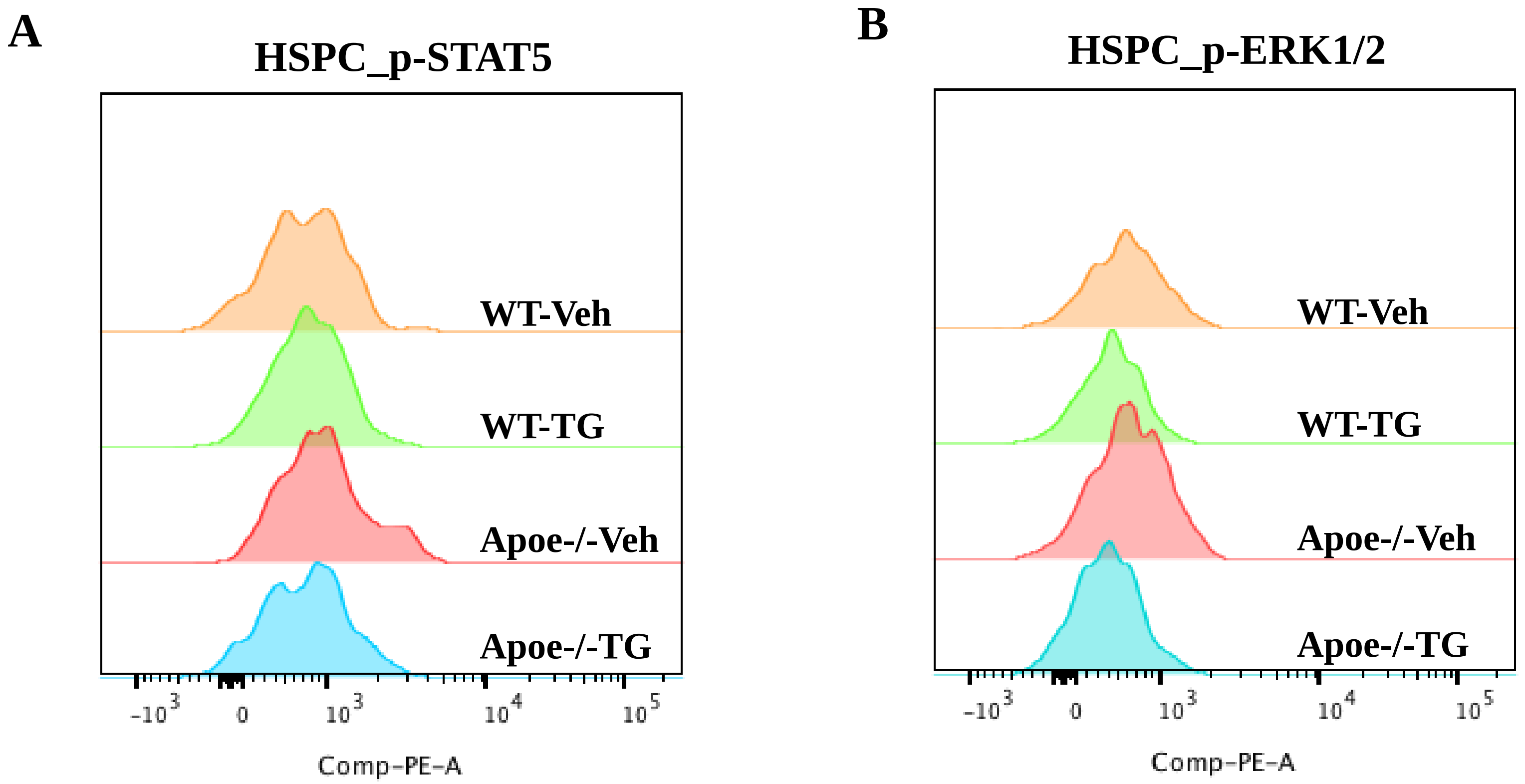

Supplemental Figure 8

Hematopoietic progenitors gating strategy

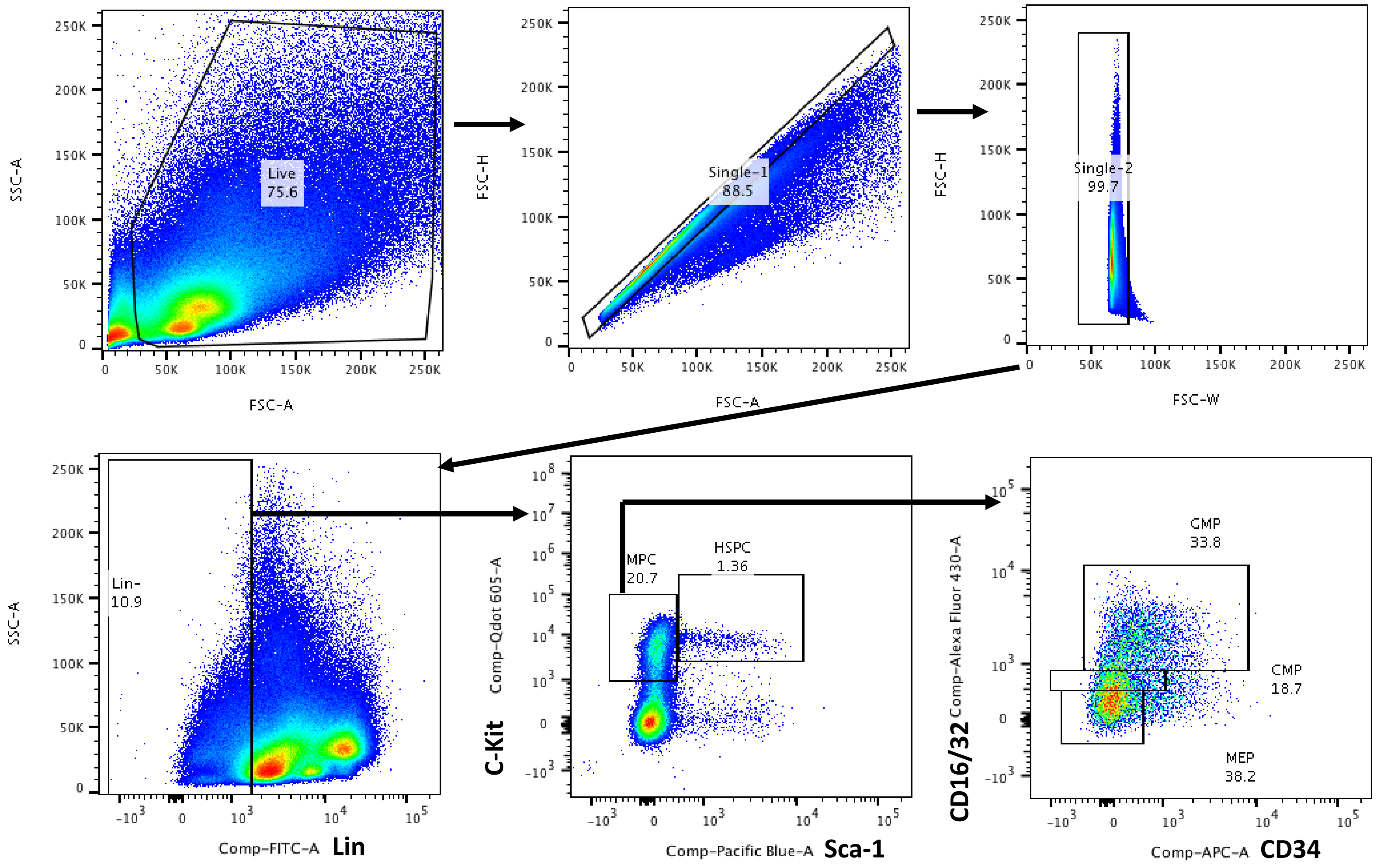

Supplement: Supplementary file 1 — Supplemental Figure 1. (A-B) Body weight after and following 9 weeks of vehicle or TG101348 (8-10 mice/group). (C) plasma total cholesterol levels and (D) HDL-cholesterol following 30 days of vehicle or TG101348 treatment (8-10 mice/group). One-way ANOVA. Data are mean ± SEM. **p<0.01, ***p<0.001. Supplemental Figure 2. Baseline levels of (A) neutrophil or (B) monocyte counts in peripheral blood (at day 0 before vehicle or TG101348 treatment) were shown. One-way ANOVA. Data are mean ± SEM. *p<0.05, **p<0.01, ***p<0.001. Supplemental Figure 3. WT and Apoe-/- mice hemoglobin and platelet counts. (A) The baseline hemoglobin concentration and (B) platelet counts in peripheral blood (at day 0 before vehicle or TG101348 treatment). (C) hemoglobin concentration and (D) platelet counts in peripheral blood following vehicle or TG101348 treatment for 30 days were shown. One-way ANOVA. Data are mean ± SEM. *p<0.05, **p<0.01, ***p<0.001. Supplemental Figure 4. Red blood cell counts following treatment with vehicle or TG101348 for 30 days. One-way ANOVA. Data are mean ± SEM. ***p<0.001. Supplemental Figure 5. Flow cytometry gating strategy of blood neutrophil and monocyte. Supplemental Figure 6. Decreased spleen weight in TG101348 treated Apoe-/- mice. Mice were fed WD for one week following by 9 weeks WD with vehicle or TG101348 treatment. (A) Absolute spleen weight and (B) spleen/body weight ratios. One-way ANOVA. Data are mean ± SEM. ***p<0.001. Supplemental Figure 7. Representative flow cytometric histograms of (A) p-STAT5 and (B) p-ERK1/2 in HSPC. Supplemental Figure 8. Flow cytometry gating strategy of hematopoietic progenitor cells. (PDF 414 kb) [file 10557_2020_6943_MOESM1_ESM.pdf]
